# Highly Pathogenic Avian Influenza A(H5Nx) Virus of Clade 2.3.4.4b Emerging in Tibet, China, 2021

**DOI:** 10.1128/spectrum.00643-22

**Published:** 2022-04-21

**Authors:** Yi Li, Xiang Li, Xinru Lv, Qiuzi Xu, Zhenliang Zhao, Siyuan Qin, Peng Peng, Fengyi Qu, Rongxiu Qin, Qing An, Meixi Wang, Zhen Zhang, Hua Luo, Xiangwei Zeng, Yulong Wang, Zhijun Hou, Heting Sun, Yajun Wang, Yu Xu, Yanbing Li, Hongliang Chai

**Affiliations:** a Northeast Forestry University, College of Wildlife and Protected Area, Harbin, China; b State Key Laboratory of Veterinary Biotechnology, Chinese Academy of Agricultural Sciences, Harbin Veterinary Research Institute, Harbin, China; c Biological Disaster Control and Prevention Center, National Forestry and Grassland Administration, Shenyang, China; Changchun Veterinary Research Institute

**Keywords:** HPAIV, H5N8, H5N1, clade 2.3.4.4b, migratory birds

## Abstract

H5N8 and H5N1 highly pathogenic avian influenza viruses (AIVs) of clade 2.3.4.4b were isolated from dead migratory birds and fecal samples collected in Tibet, China, in May 2021. Phylogenetic analyses showed that the viruses isolated in this study may have spread from wintering or stopover grounds of migratory birds in South Asia. We monitored two disparate clade 2.3.4.4b H5Nx viruses in migratory birds in Tibet during their breeding season. The data revealed that breeding grounds may exhibit a potential pooling effect among avian influenza viruses in different migratory populations.

**IMPORTANCE** In this study, 15 H5N8 and two H5N1 highly pathogenic avian influenza viruses of clade 2.3.4.4b were isolated from dead migratory birds and fecal samples in Tibet, China. Isolates of H5N1 virus of clade 2.3.4.4b have been rarely reported in China. Our findings highlight that breeding grounds may exhibit a potential pooling effect among avian influenza viruses (AIVs) in different migratory populations. In addition to intensification of the surveillance of AIVs in migratory birds in Tibet, China, international cooperation should be strengthened.

## OBSERVATION

Clade 2.3.4 H5 highly pathogenic avian influenza viruses (HPAIVs) have undergone reassortment with other neuraminidase segments since 2010 (1), which has led to the emergence of clade 2.3.4.4b H5Nx viruses (e.g., H5N1, H5N2, H5N3, H5N4, H5N5, H5N6, and H5N8) (2) (https://www.offlu.org/wp-content/uploads/2021/11/OFFLU-November2021-Final.pdf). Significantly, H5N8 has gradually become the dominant strain across the world, resulting in three waves of intercontinental epidemics (3). In early 2014, clade 2.3.4.4 HPAIV H5N8 induced outbreaks in South Korea followed by extensive transmission throughout eastern Asia, Europe, Russia, and North America via migratory birds (4). In 2016, the novel H5N8 virus obtained gene segments from the Eurasian low-pathogenicity avian influenza viruses (LPAIVs) reported in Eurasia (5, 6). From 2020 to 2021, multiple outbreaks of H5N8 viruses were reported in many European and Asian countries (7–9). While clade 2.3.4.4b HPAIV H5N8 was reported in humans in February 2021, no evidence of human-to-human transmission was found (10). The H5N8 virus had also undergone reassortment with other wild bird influenza viruses to form new strains of HPAIV H5N1 since the autumn of 2020 (11). It subsequently continued to be prevalent in Africa, Europe, and Asia. In the present study, we describe the genetic characterizations of clade 2.3.4.4b H5N8 and H5N1 HPAIVs causing outbreaks among migratory birds in Tibet, China, 2021.

In May 2021, a number of bar-headed geese (Anser indicus) and brown-headed gulls (Larus brunnicephalus Jerdon) were found dead in the Naqu region in northern Tibet, China (31.9161N, 91.5322E to 31.3028N, 91.7667E). The Naqu region is one of the most important breeding grounds for bar-headed geese and brown-headed gulls that overwinter in southern Tibet and South Asia (12). The organs (trachea, liver, lung, pancreas, kidney, spleen, and rectum) or swabs (nasopharyngeal and cloacal) from dead birds were collected. Nearby fecal samples were also collected (see Table S1 in the supplemental material). Next, virus isolation and genome sequencing were performed as previously described (13). A total of 15 H5N8 viruses (TB-H5N8) and two H5N1 viruses (TB-H5N1) were isolated from the samples. All viral genome sequences were deposited into the GISAID EpiFlu database (https://www.gisaid.org) (Table S2).

All eight gene segments of TB-H5N8 displayed a high nucleotide identity of 99.1% to 100.0%. TB-H5N1 viruses shared a 99.5% to 100.0% nucleotide identity. TB-H5N8 and TB-H5N1 shared a high level of nucleotide similarity in the HA and M genes (98.6% to 99.4% and 99.5% to 99.6%, respectively); however, there was a lower level of sequence identity in the other internal gene segments (PB2, 93.0% to 93.2%; PB1, 93.5% to 93.7%; PA, 94.6% to 94.7%; NS, 91.4% to 91.8%; NP, 95.6% to 95.7%). The hemagglutinin cleavage site of 17 H5Nx viruses, sequenced as REKRRKR*GLF, indicated that they were HPAIVs. All of the receptor binding sites of the isolates at positions 222 to 224 (H5 numbering) were QRG; however, substitutions S123P, S133A, and T156A were identified in the HA gene, suggesting an increased affinity to human-like (α2,6-linked sialic acid) receptors (14). The Q591, E627, and D701 residues in the PB2 protein suggest that these viruses have not yet adapted to mammalian hosts (15) (see Table S3).

**FIG 1:**
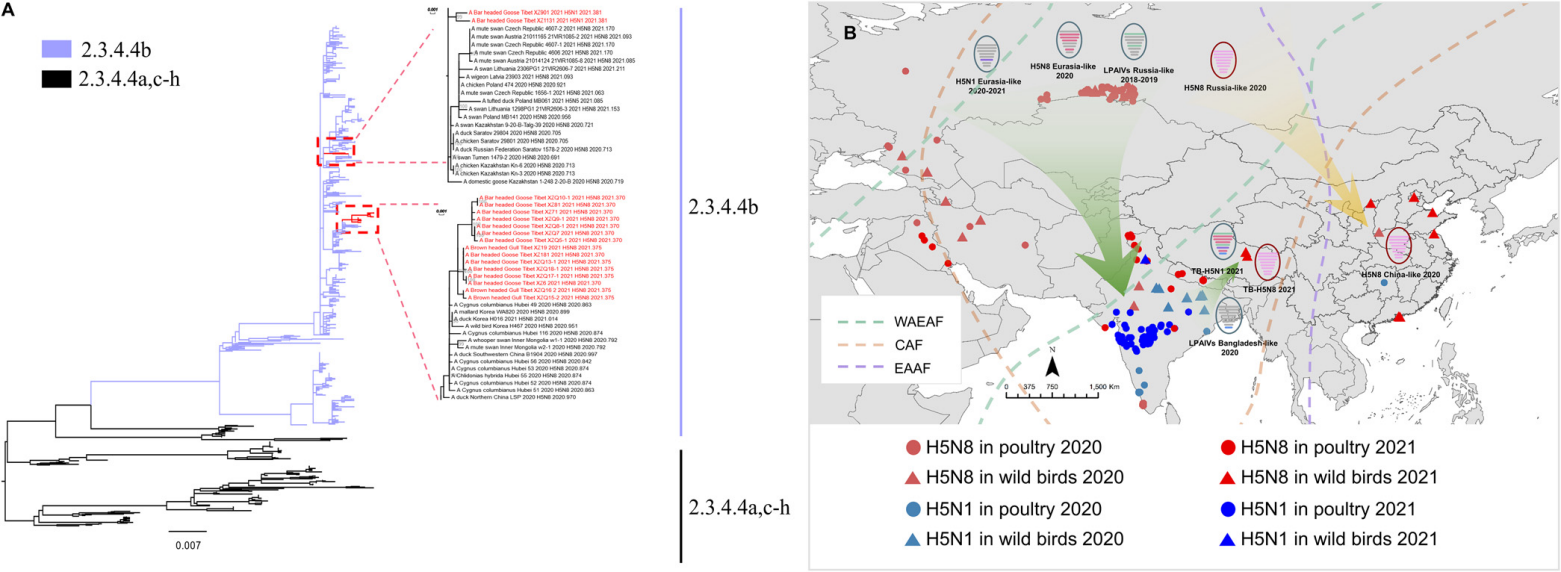
Phylogenetic analysis of HA and hypothetical evolution and pathway of Tibet H5Nx virus spread. (A) Maximum-likelihood phylogenetic tree of the HA genes of H5Nx viruses isolated in Tibet, China, and compared with the clade 2.3.4.4 reference isolates. The Tibet H5Nx isolates are marked in red. Bootstrap supports higher than 90% are indicated. The scale bar indicates the nucleotide substitutions per site. (B) Hypothetical evolution and pathway of influenza (H5Nx) virus spread from Tibet, China. Confirmed highly pathogenic avian influenza events in Central Asia, South Asia, China, and Russia were available through the OIE between 2020 and May 2021. WAEAF, West Asian-East African Flyway; CAF, Central Asian Flyway; EAAF, East Asian-Australasian Flyway. The map in Fig. 1B obtained from a website (https://365psd.com).

The HA phylogenic tree indicated that all H5 isolates belonged to clade 2.3.4.4b (Fig. 1A). Our analyses suggested that the TB-H5N8 isolates were closely related to clade 2.3.4.4b HPAIV H5N8 circulating in wild birds in China in 2020, which originated in Russia (16). The closest genetic relatives of TB-H5N1 in the NS gene segment were found to be LPAIVs. And, the NS gene of TB-H5N1 shared the highest nucleotide identity with that of the H10 viruses isolated in Bangladesh in ducks in 2020 (98.7% to 98.8%). The NP and PB2 genes shared the highest nucleotide identity with A/gadwall/Chany/893/2018(H3N8) (99.1%) and A/mallard/Novosibirsk region/1894k/2019(H4N6) (98.7%), respectively. The other five gene segments were clustered with the Eurasian clade 2.3.4.4b H5Nx HPAIVs from 2020 to 2021. A molecular dating analysis using BEAST 1.8.4 indicated that the ancestor of the TB-H5N8 isolates had circulated from November 2020 to February 2021. The generation of TB-H5N1 isolates appears to have been a complex process and was likely completed prior to May 2021.

### Discussions and conclusions.

Here, we report the emergence of clade 2.3.4.4b H5N8 and H5N1 HPAIVs in Tibet, China, which was accompanied by serious disease in wild migratory birds.

The maximum likelihood phylogenetic trees showed that eight gene segments of TB-H5N8 clustered with H5N8 HPAIVs in China in 2020, which originated in Russia (see Fig. S1 in the supplemental material). The maximum clade credibility phylogenetic trees of eight genes revealed that the ancestral viruses of TB-H5N8 were likely circulating from November 2020 to February 2021, during the overwintering and initiation of spring migration (see Fig. S2 and Table S4). The outbreak occurred in May, which is the peak season for these migratory birds to breed in north Tibet. Since there are no farms or backyard poultry located near the outbreak sites, we speculated that TB-H5N8 was the most likely continuously circulate in wintering or stopover grounds. Thus, the complex migration patterns of bar-headed geese or brown-headed gulls are likely to cause an outbreak of TB-H5N8.

Since H5N1 virus of clade 2.3.4.4b was reported in 2020 (11), it has subsequently continued to be prevalent in Africa, Europe, and Asia. The closest genetic relatives of the NS gene segment of TB-H5N1 were LPAIVs in Bangladesh in 2020. The NP and PB2 genes shared the highest nucleotide identity with the LPAIVs in Russia. The other five gene segments of TB-H5N1 clustered with the Eurasian clade 2.3.4.4b H5Nx viruses from 2020 to 2021. Among them, only NA, PA, and NP were clustered with clade 2.3.4.4b H5N1 from the Netherlands in 2020 (see Fig. S1). Therefore, TB-H5N1 and H5N1 in the Netherlands in 2020 had a different evolutionary trajectory. The estimation of the most recent common ancestor of TB-H5N1 indicated that the isolates might have entered Tibet between January and May 2021 when the bar-headed geese migrated in the spring and bred (see Fig. S2 and Table S4). The migration routes of bar-headed geese along the Central Asian flyway have been identified (12). In the spring, a large number of migrating birds arrive at their breeding grounds in northern Tibet from their wintering grounds in southern Tibet and South Asia (i.e., India, Nepal, and Bangladesh). Although no sequences for HPAIV H5N1 in South Asia have been submitted to GISAID or GenBank, in January 2021, the World Organization for Animal Health (OIE) reported sporadic outbreaks of H5N1 in bar-headed geese in India (https://wahis.oie.int). As a consequence, we inferred that the reassortment between LPAIVs and clade 2.3.4.4b H5Nx HPAIVs occurred in wintering or stopover grounds in South Asia (i.e., India). Subsequently, the TB-H5N1 viruses spread to Tibet during spring migration through migratory birds (Fig. 1B). The long branch lengths from some segments of TB-H5N1 to their closest relatives suggested that these viruses had been circulating undetected for the intervening period and complex reassortment may have occurred. Therefore, it is particularly important to strengthen the active surveillance of avian influenza in wintering and stopover regions of bar-headed geese (e.g., Indian Peninsula and Bay of Bengal region). It is necessary to strengthen the international cooperation related to influenza surveillance, which will facilitate viral tracing and an early warning system.

Tibet, located mainly on the Central Asian Flyways, which is the first stop for the wintering birds of the Indian Peninsula and Bay of Bengal region migrating to China. AIVs are carried by wild migratory waterfowl across migratory flyways. To determine the strains of circulating AIVs that may pose a risk to poultry and humans, regular surveillance studies of wild birds must be performed. However, due to limitations of a high altitude and harsh natural environment, active surveillance of avian influenza among wild birds in the region remains incomplete. Therefore, there is a need to carry out continued active surveillance of avian influenza activity before and after spring and autumn migration in Tibet as an early warning system.

### Data availability.

All viral genome sequences have been deposited into the GISAID EpiFlu database (https://www.gisaid.org) under the GISAID accession numbers EPI_ISL_8215653, EPI_ISL_8215654, EPI_ISL_8215655, EPI_ISL_8215656, EPI_ISL_8215657, EPI_ISL_8215658, EPI_ISL_8215659, EPI_ISL_8215660, EPI_ISL_8215661, EPI_ISL_8215662, EPI_ISL_8215663, EPI_ISL_8215684, EPI_ISL_8215685, EPI_ISL_8215686, EPI_ISL_8215687, EPI_ISL_8215688, and EPI_ISL_8215689.
