# Supplementary material for: Highly Pathogenic Avian Influenza A(H5Nx) Virus of Clade 2.3.4.4b Emerging in Tibet, China, 2021
Source: Microbiol Spectr. 2022 Apr 21;10(3):e00643-22. doi: 10.1128/spectrum.00643-22 (PMC9241900; doi:10.1128/spectrum.00643-22)

Highly Pathogenic Avian Influenza A(H5Nx) virus of Clade 2.3.4.4b Emerging in Tibet, China, 2021

Appendix

Materials and Methods

Phylogenetic Analysis

The H5 gene of global HPAI H5 (2019–2021) viruses available in GISAID and the representative strains of 2.3.4.4a–h were also download to build the H5 phylogenetic tree. A BLASTn search was performed against sequences in the GISAID database. The top 100 BLASTn hits of eight sequences of each isolates were also download. Sequences were aligned using MAFFT (1) implemented in PhyloSuite 1.2.2 (2). Duplicated and information-missing sequences were removed. Maximum likelihood phylogenies were generated using IQ-TREE (3) under the best-fit substitution model for 10000 ultrafast bootstraps (4). The best-fit substitution model was selected using the Bayesian information criterion by ModelFinder (5) implemented in PhyloSuite 1.21(6). Visualization and annotation of the trees were performed by Evolview (http://www.evolgenius.info/evolview).

Molecular Dating

We first carried out a root-to-tip distance analysis for viral segments by TempEst v1.5.3 to make sure there was a temporal signal (7). Then, the time of the most recent common ancestor (tMRCA) for each segment was estimated by Bayesian analysis using BEAST version 1.8.4 (8). The best-fit substitution model was chosen as described above. We specified an uncorrelated lognormal relaxed clock and constant size tree prior for each segment (9–10). A Markov chains Monte Carlo (MCMC) method was used with 50 million chain lengths to draw inference under this model. All effective sample size values in the results were greater than 200. Multiple runs were combined using LogCombiner v1.8.4 (http://beast.community/logcombiner). Maximum clade credibility trees were combined after removing initial 10% burn-in and then reconstructed using TreeAnnotator v1.8.4 (http://beast.community/treeannotator). Visualization and annotation of the trees were

performed by FigTree 1.4.3 (http://tree.bio.ed.ac.uk/software/figtree/).

References

1. Katoh K, Standley DM. 2013. MAFFT multiple sequence alignment software version 7: improvements in performance and usability. Mol Biol Evol 30(4):772–780. https://doi.org/10.1093/molbev/mst010

2. Zhang D, Gao F, Jakovlić I, Zou H, Zhang J, Li WX, Wang GT. 2020. PhyloSuite: An integrated and scalable desktop platform for streamlined molecular sequence data management and evolutionary phylogenetics studies. Mol Ecol Resour 20(1):348–355. https://doi.org/10.1111/1755-0998.13096

3. Nguyen LT, Schmidt HA, von Haeseler A, Minh BQ. 2015. IQ-TREE: a fast and effective stochastic algorithm for estimating maximum-likelihood phylogenies. Mol Biol Evol 32(1):268–274. https://doi.org/10.1093/molbev/msu300

4. Minh BQ, Nguyen MA, von Haeseler A. 2013. Ultrafast approximation for phylogenetic bootstrap. Mol Biol Evol 30(5):1188–1195. https://doi.org/10.1093/molbev/mst024

5. Kalyaanamoorthy S, Minh BQ, Wong TKF, von Haeseler A, Jermiin LS. 2017. ModelFinder: fast model selection for accurate phylogenetic estimates. Nat Methods 14(6):587-589. https://doi.org/10.1038/nmeth.4285

6. Zhang D, Gao F, Jakovlić I, Zou H, Zhang J, Li WX, Wang GT. 2020. PhyloSuite: An integrated and scalable desktop platform for streamlined molecular sequence data management and evolutionary phylogenetics studies. Mol Ecol Resour 20(1):348–355. https://doi.org/10.1111/1755-0998.13096

7. Rambaut A, Lam TT, Max Carvalho L, Pybus OG. 2016. Exploring the temporal structure of heterochronous sequences using TempEst (formerly Path-O-Gen). Virus evolution 2(1):vew007. https://doi.org/10.1093/ve/vew007

8. Drummond AJ, Rambaut A. 2007. BEAST: Bayesian evolutionary analysis by sampling trees. BMC Evol Biol 7:214. https://doi.org/10.1186/1471-2148-7-214

9. Drummond AJ, Ho SY, Phillips MJ, Rambaut A. 2006. Relaxed phylogenetics and dating with confidence. PLoS Biol 4(5):e88. https://doi.org/10.1371/journal.pbio.0040088

10. Kingman, J. F. C. 1982. The coalescent. Stoch Process Their Appl 13:235–248. https://doi.org/10.1016/0304-4149(82)90011-4

Appendix Table 1. Information of wild bird samples collected in Tibet, May 2021.

location	Collection date	Types of samples (positive for AIV)		
		Faeces	Organs	Swabs
Anduo county	2021/5/15	364 (4)	12 (5) [BHGS]*	0
Sene District	2021/5/17	403	2 (2) [BHGL]<br>4 (3) [BHGS]	8 (1) [BHGL]
Baingoin County	2021/5/18	90	0	0
Xainza County	2021/5/20	558 (2)	0	0
Total	2021/5/15-2021/5/20	1415 (6)	18 (10)	8 (1)

*Species: BHGS, Bar-headed goose; BHGL, Brown-headed gull.

Appendix Table 2. Information of the 17 H5Nx viruses isolated in Tibet, May 2021.

GISAID accession no.	Isolate name	Collection date	Types of samples
EPI_ISL_8215653	A/Bar-headed Goose/Tibet/XZQ5-1/2021(H5N8)	2021-May-15	organs
EPI_ISL_8215654	A/Bar-headed Goose/Tibet/XZQ7/2021(H5N8)	2021-May-15	organs
EPI_ISL_8215655	A/Bar-headed Goose/Tibet/XZQ8-1/2021(H5N8)	2021-May-15	organs
EPI_ISL_8215656	A/Bar-headed Goose/Tibet/XZQ9-1/2021(H5N8)	2021-May-15	organs
EPI_ISL_8215657	A/Bar-headed Goose/Tibet/XZQ10-1/2021(H5N8)	2021-May-15	organs
EPI_ISL_8215658	A/Bar-headed Goose/Tibet/XZ6/2021(H5N8)	2021-May-15	faeces
EPI_ISL_8215659	A/Bar-headed Goose/Tibet/XZ71/2021(H5N8)	2021-May-15	faeces
EPI_ISL_8215660	A/Bar-headed Goose/Tibet/XZ81/2021(H5N8)	2021-May-15	faeces
EPI_ISL_8215661	A/Bar-headed Goose/Tibet/XZ181/2021(H5N8)	2021-May-15	faeces
EPI_ISL_8215662	A/Bar-headed Goose/Tibet/XZQ13-1/2021(H5N8)	2021-May-17	organs
EPI_ISL_8215663	A/Brown-headed Gull/Tibet/XZQ15-2/2021(H5N8)	2021-May-17	organs
EPI_ISL_8215684	A/Brown-headed Gull/Tibet/XZQ16-2/2021(H5N8)	2021-May-17	organs
EPI_ISL_8215685	A/Bar-headed Goose/Tibet/XZQ17-1/2021(H5N8)	2021-May-17	organs
EPI_ISL_8215686	A/Bar-headed Goose/Tibet/XZQ18-1/2021(H5N8)	2021-May-17	organs
EPI_ISL_8215687	A/Brown-headed Gull/Tibet/XZ19/2021(H5N8)	2021-May-17	swabs
EPI_ISL_8215688	A/Bar-headed Goose/Tibet/XZ901/2021(H5N1)	2021-May-20	faeces
EPI_ISL_8215689	A/Bar-headed Goose/Tibet/XZ1131/2021(H5N1)	2021-May-20	faeces

Appendix Table 3. Key molecular markers of the H5Nx viruses isolated in Tibet, May 2021.

Protein	Position		human-H5N8	TB-H5N8	TB-H5N1
H5 (H5 numbering)	Cleavage site		REKRRKRGLFGAI	REKRRKRGLFGAI	REKRRKRGLFGAI
	222-224	increased binding to avian-like (α 2–3–SA) receptors	QRG	QRG	QRG
	Thr156Ala	increased binding to human-like (α 2–6–SA) receptors	A	A	A
	Thr188Ile	increased binding to human-like (α 2–6–SA) receptors	T	I	T
	S123P	increased binding to human-like (α 2–6–SA) receptors	P	P	P
	S133A	increased binding to human-like (α 2–6–SA) receptors	A	A	A
PB2	Q591	not yet adapted to mammalian hosts.	Q	Q	Q
	E627	not yet adapted to mammalian hosts.	E	E	E
	D701	not yet adapted to mammalian hosts.	D	D	D
M1	N30D	increase virulence to mammals	D	D	D
	I43M	increase virulence to mammals	M	M	M
	T215A	increase virulence to mammals	A	A	A
NS	P42S	increase virulence to mammals	S	S	S
	80-84 deletion	increase virulence to mammals	√	√	√
	L98F	increase virulence to mammals	F	F	F
	I101M	increase virulence to mammals	M	M	M

Appendix Table 4. Time signal analysis and Time of the most recent common ancestor (tMRCA) of each segment of the H5Nx viruses isolated in Tibet, May 2021.

**Segment**	**Correlation Coefficien**	**Most recent common ancestor**	**tMRCA**	**95% HPD interval**	**Posterior Probability**
PB2	0.9059	TB-H5N8	Nov.2020	[Sept.2020, Jan.2021]	1
		TB-H5N1	Mar.2021	[Dec.2020, May.2021]	1
PB1	0.5744	TB-H5N8	Jan.2021	[Dec.2020, Feb.2021]	1
		TB-H5N1	Apr.2021	[Mar.2021, May.2021]	1
PA	0.7484	TB-H5M8	Dec.2020	[Oct.2020, Feb.2021]	0.9726
		TB-H5N1	Apr.2021	[Feb.2021, May.2021]	1
NS	0.9281	TB-H5N8	Dec.2020	[Oct.2020, Feb.2021]	0.9997
		TB-H5N1	Mar.2021	[Jan.2021, May.2021]	1
NP	0.7435	TB-H5N8	Dec.2020	[Oct.2020, Feb.2021]	0.9991
		TB-H5N1	Apr.2021	[Feb.2021, May.2021]	1
M	0.7597	TB-H5N8	Feb.2021	[Dec.2020, Mar.2021]	0.8489
		TB-H5N1	May.2021	[Apr.2021, May.2021]	0.9991
N8	0.6792	TB-H5N8	Jan.2021	[Dec.2020, Mar.2021]	0.911
N1	0.9713	TB-H5N1	Apr.2021	[Feb.2021, May.2021]	1
H5	0.8819	TB-H5N8	Jan.2021	[Dec.2020, Mar.2021]	1
		TB-H5N1	Jan.2021	[Nov.2020, Apr.2021]	1

Appendix Figure 1. Maximum-likelihood phylogenetic trees. Our Tibet H5Nx isolates are marked in red. A UFBoot support values >90 was shown. Segments shown: A) polymerase basic 2 (PB2); B) polymerase basic 1(PB1); C) polymerase acidic (PA); D) nucleoprotein (NP); E) nonstructural (NS); F) matrix (M); G) neuraminidase-N8 subtype (N8); H) neuraminidase-N1 subtype (N1). Scale bar indicates nucleotide substitutions per site. Human case of H5N8 is shown in blue box.

Appendix Figure 2. Maximum clade credibility phylogenetic trees of each gene segment of the viruses associated with the H5Nx viruses isolated in Tibet, May 2021. Tibet H5Nx isolates are shown in red. Human case of H5N8 is shown in blue box. The horizontal bars indicate the 95% highest posterior density (HPD) intervals of tMRCA. Segments shown are: A) polymerase basic 2 (PB2); B) polymerase basic 1(PB1); C) polymerase acidic (PA); D) nucleoprotein (NP); E) nonstructural (NS); F) matrix (M); G) neuraminidase-N8 subtype (N8); H) neuraminidase-N1 subtype (N1); I) hemagglutinin (HA).

Appendix Figure 1A. PB2

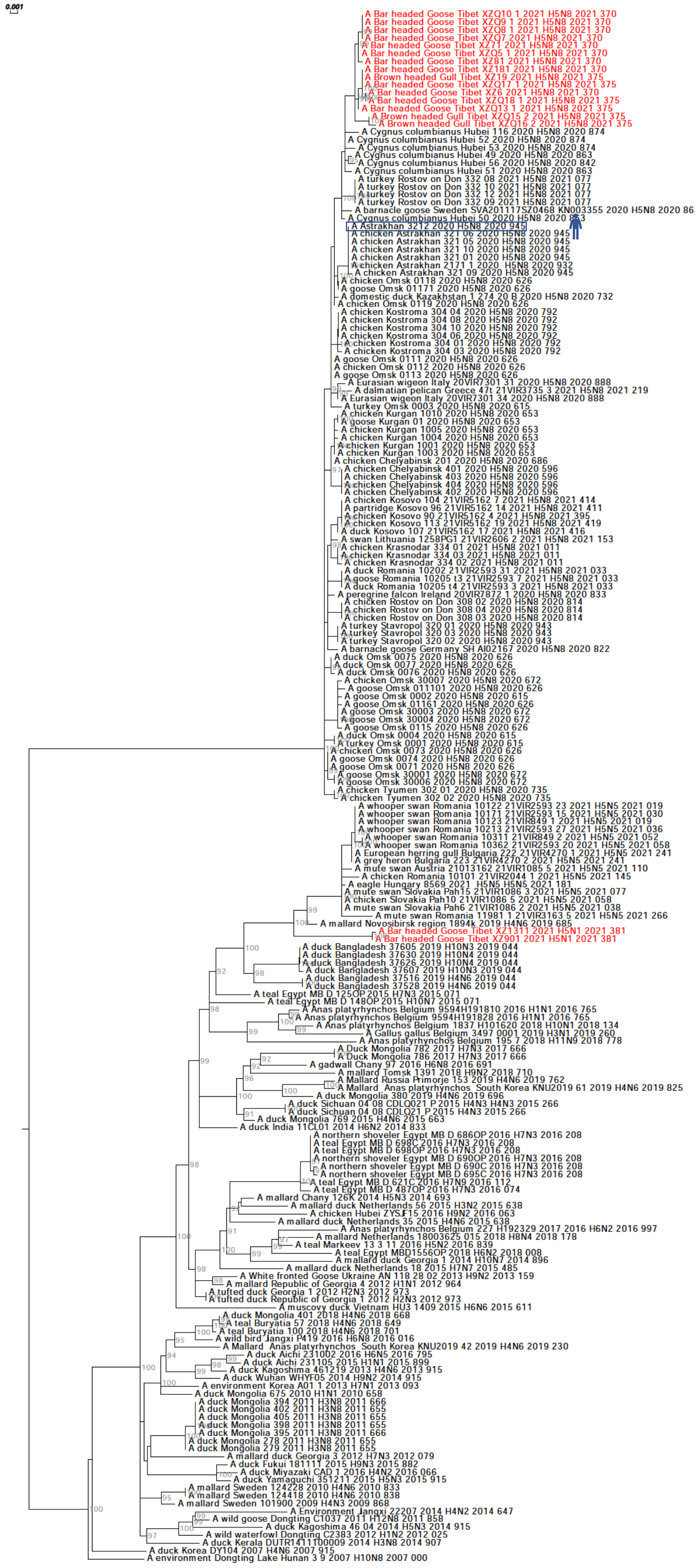

Appendix Figure 1B. PB1

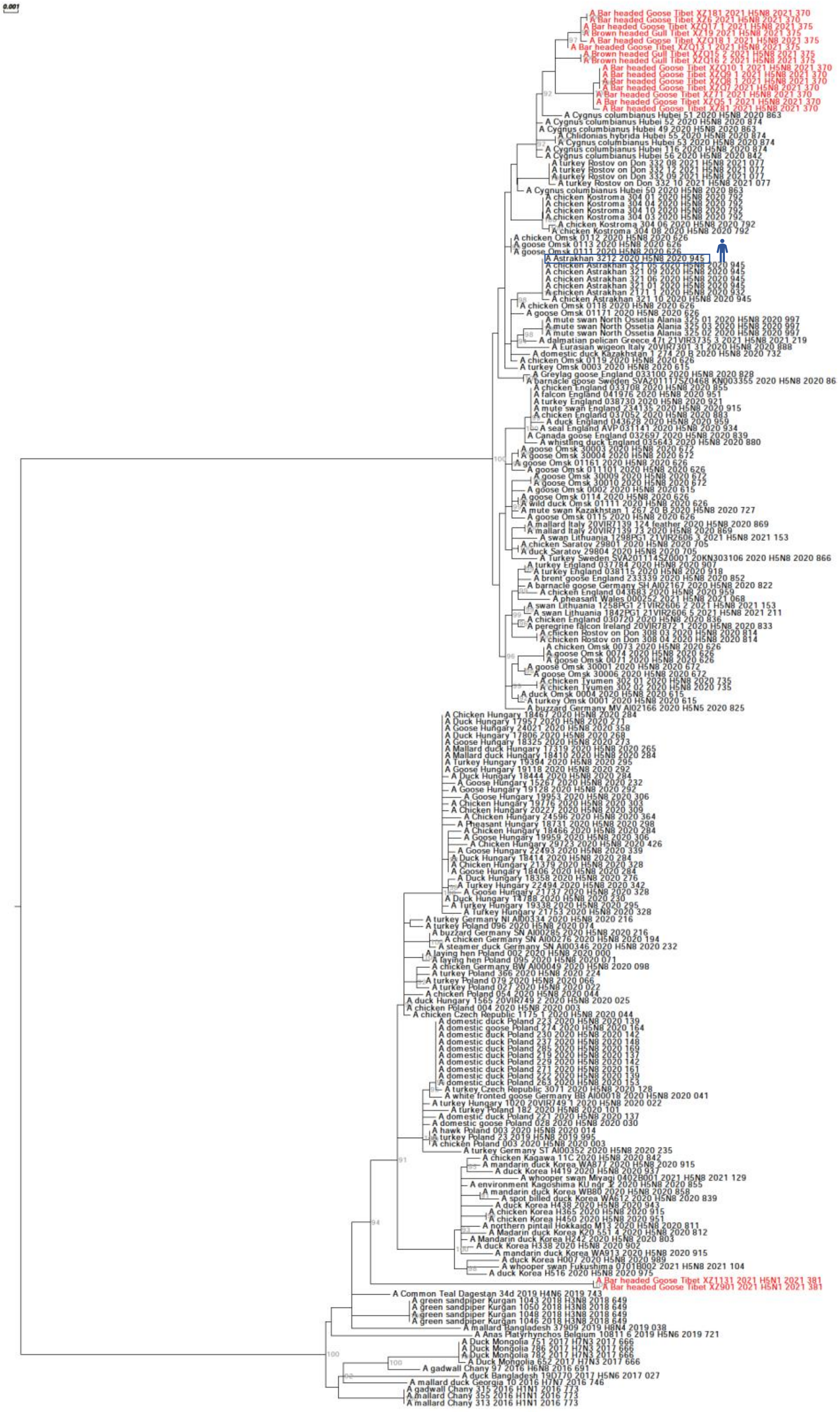

Appendix Figure 1C. PA

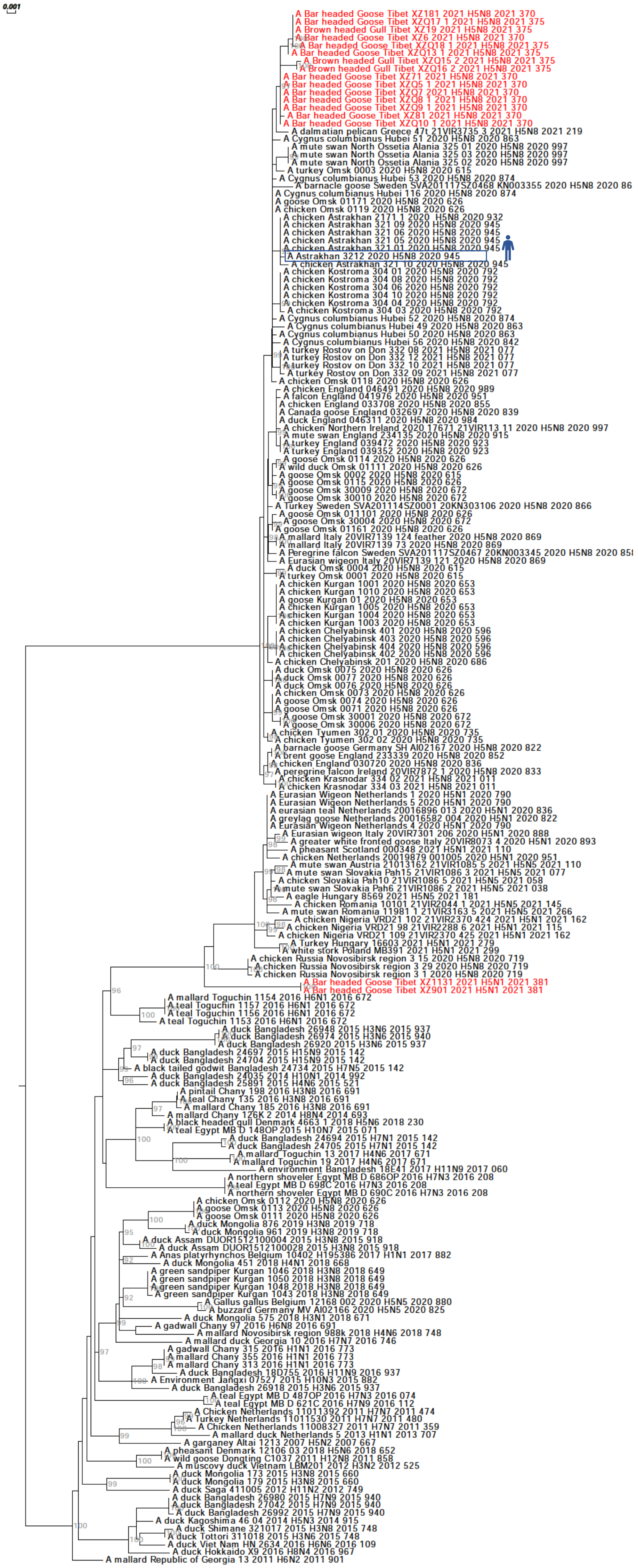

Appendix Figure 1D. NP

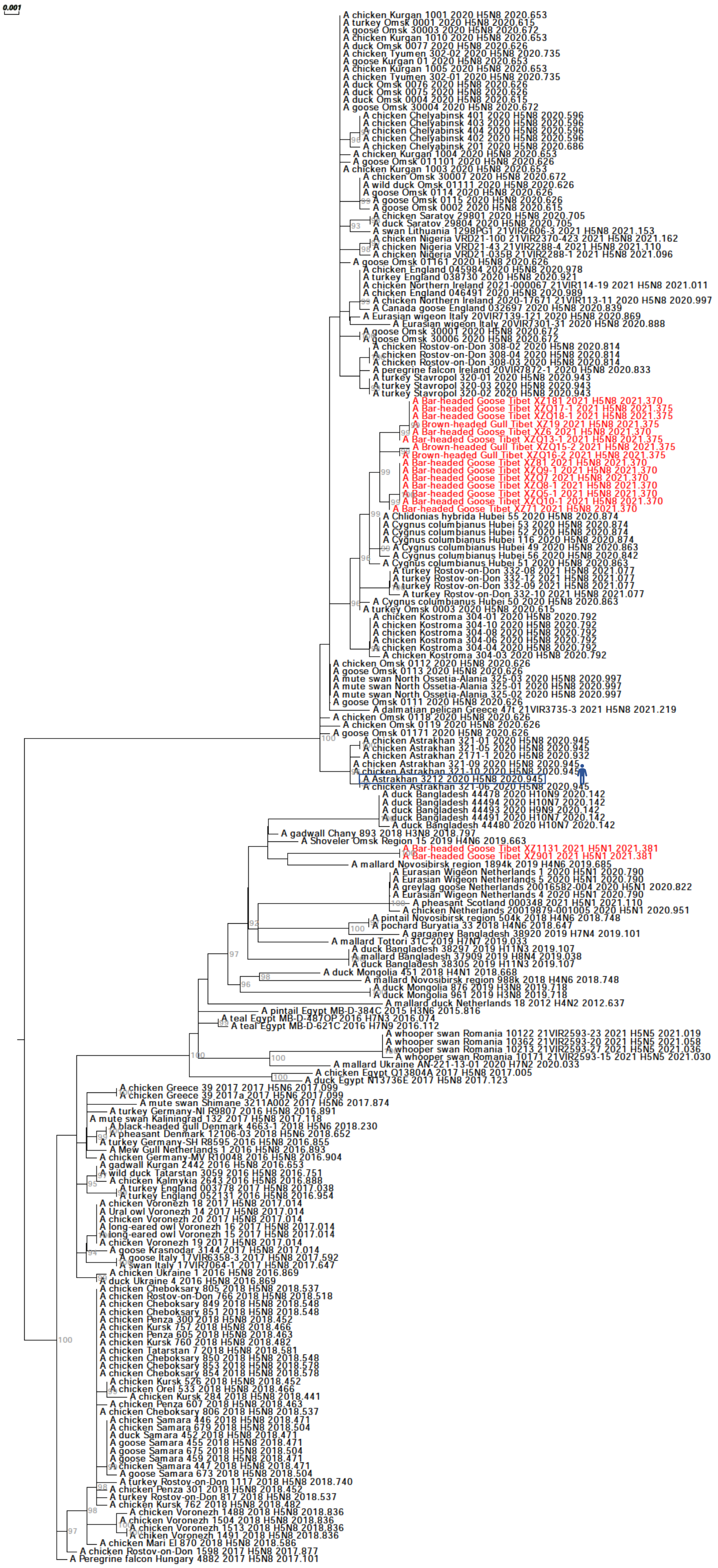

Appendix Figure 1E. NS

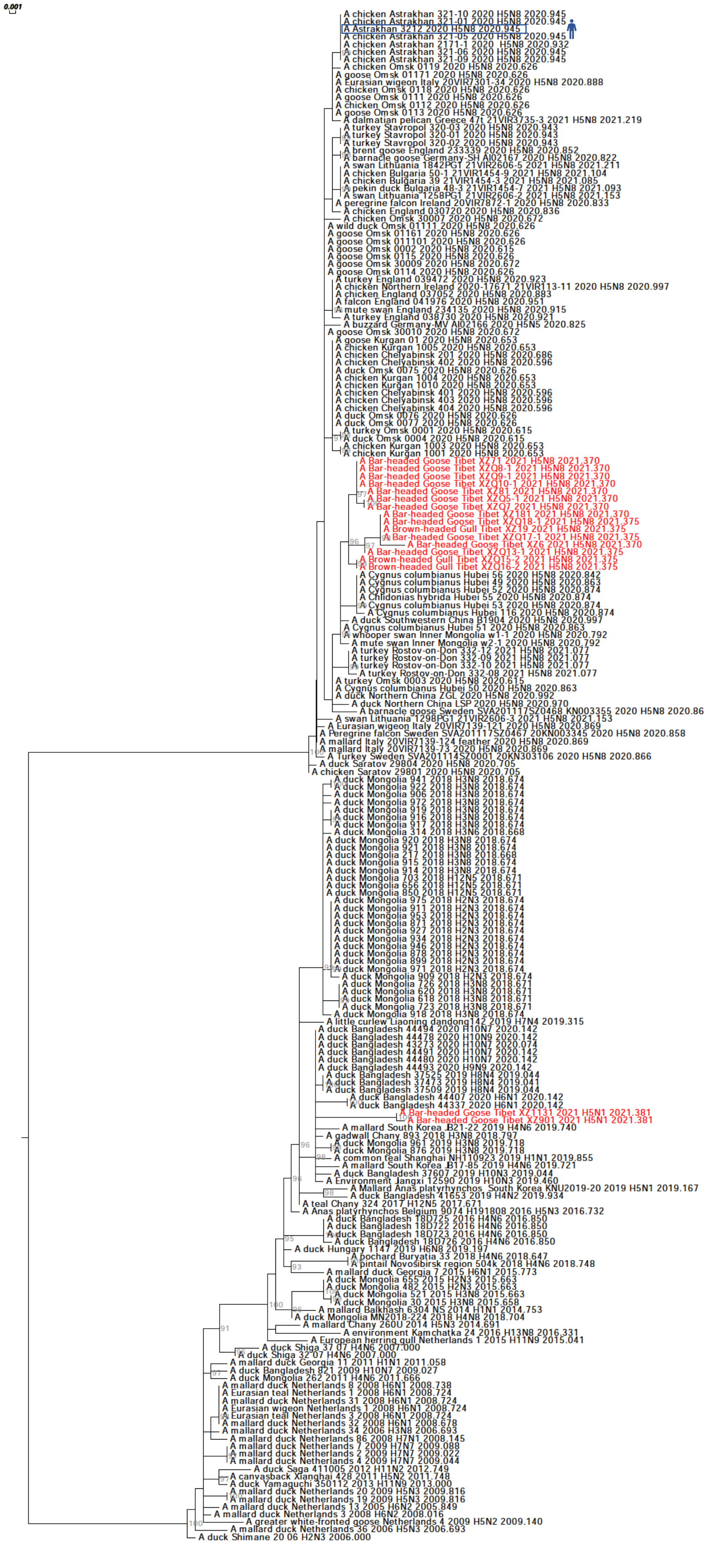

Appendix Figure 1F. M

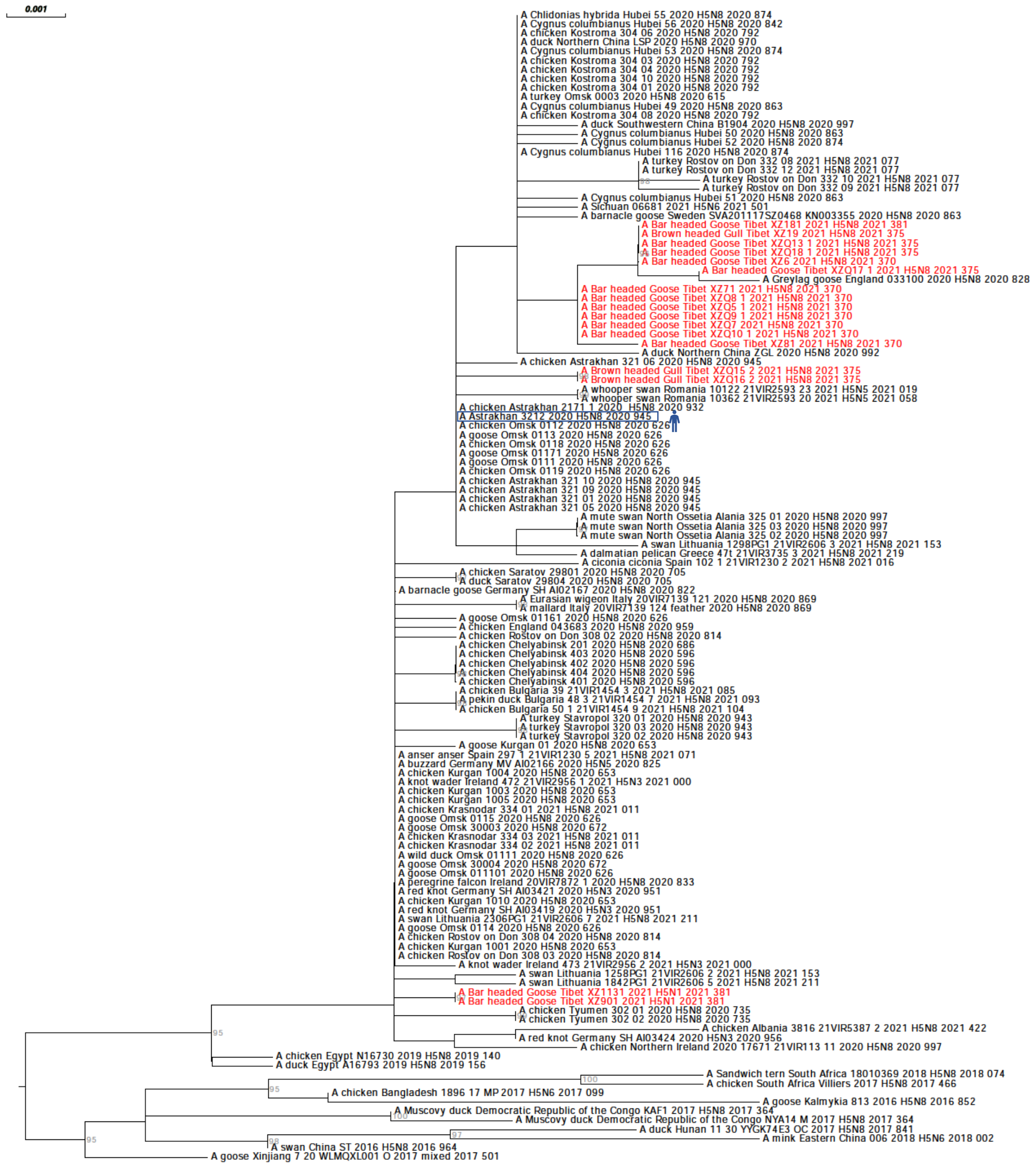

Appendix Figure 1G. N8

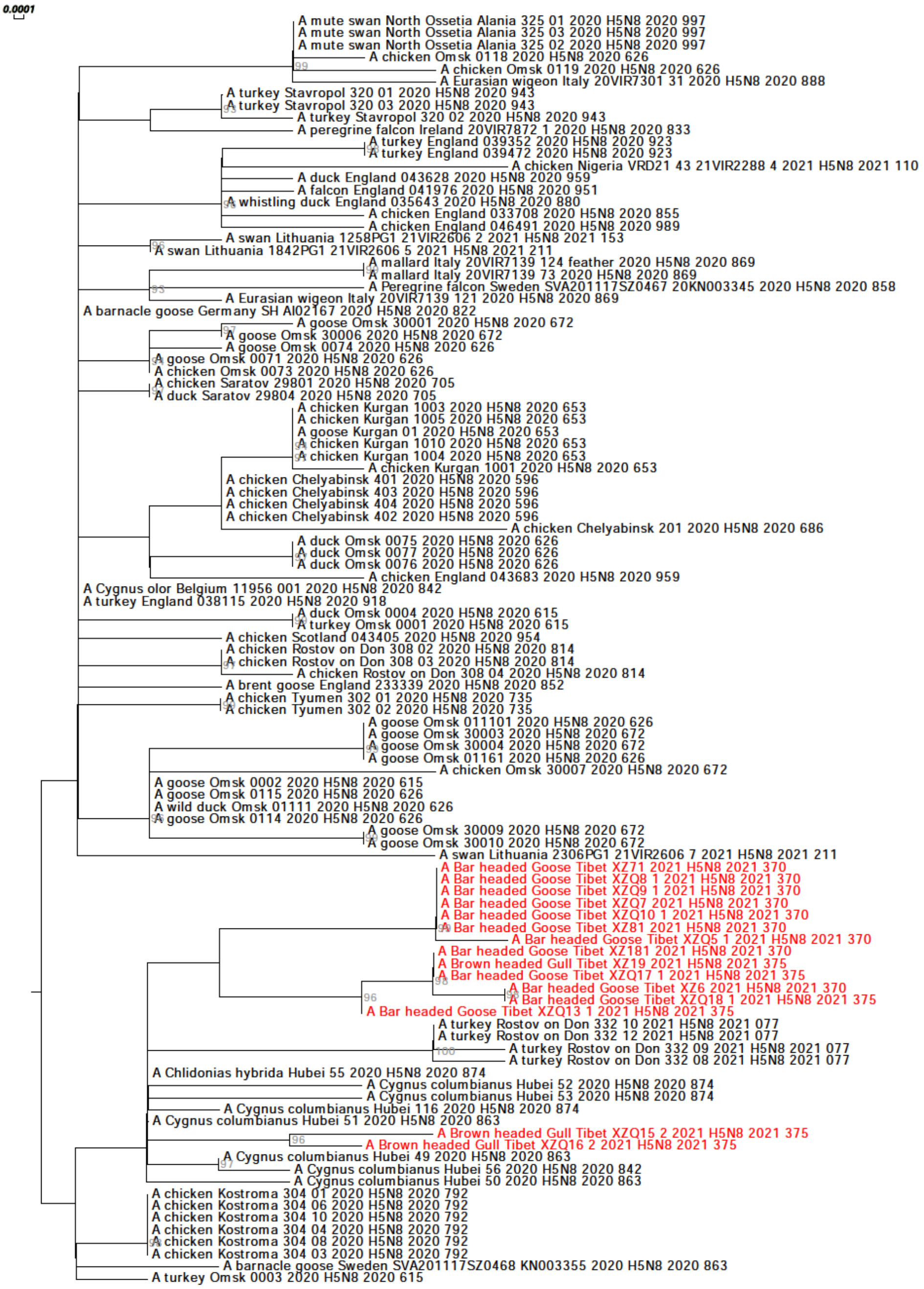

Appendix Figure 1H. N1

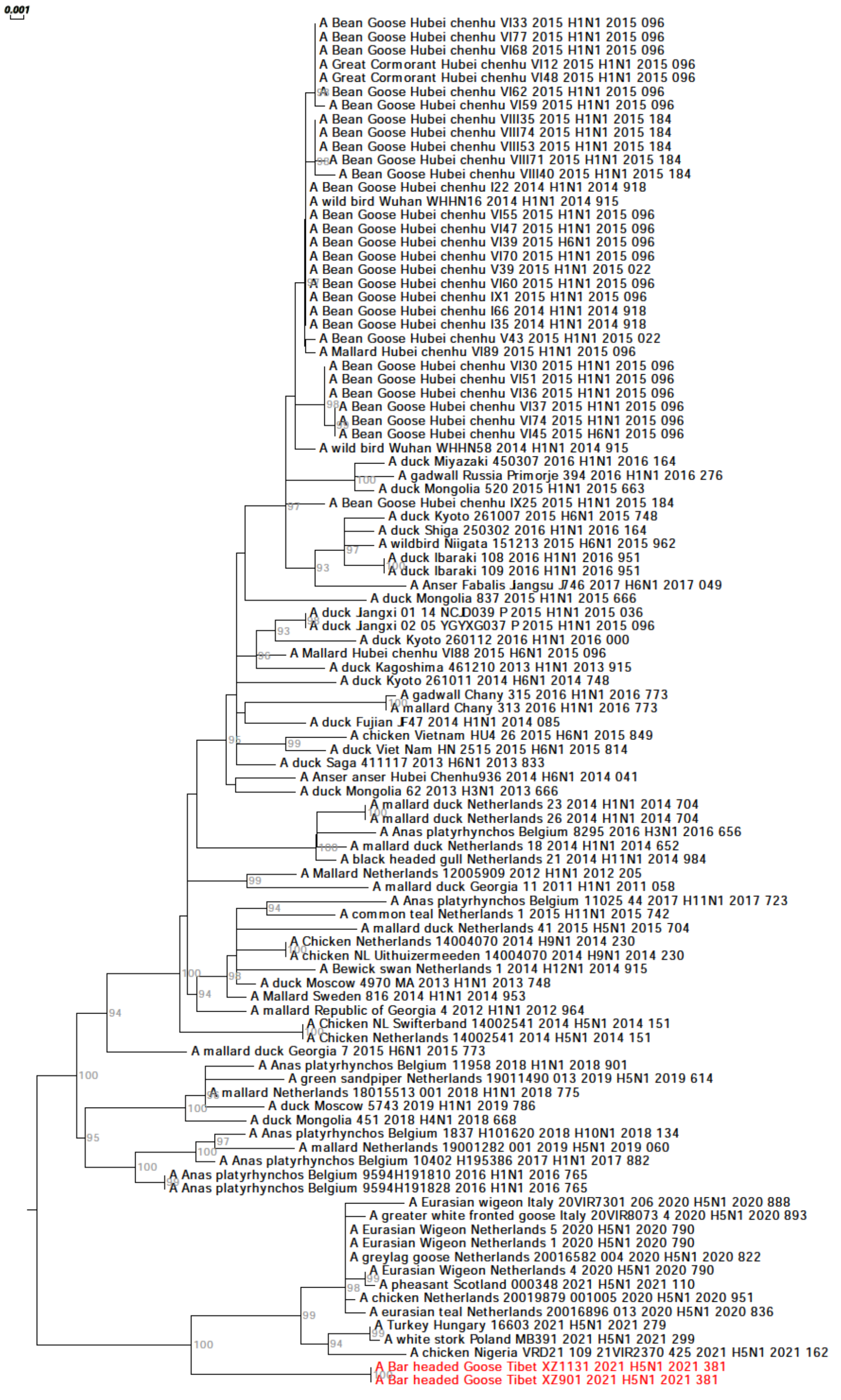

Appendix Figure 2A. PB2

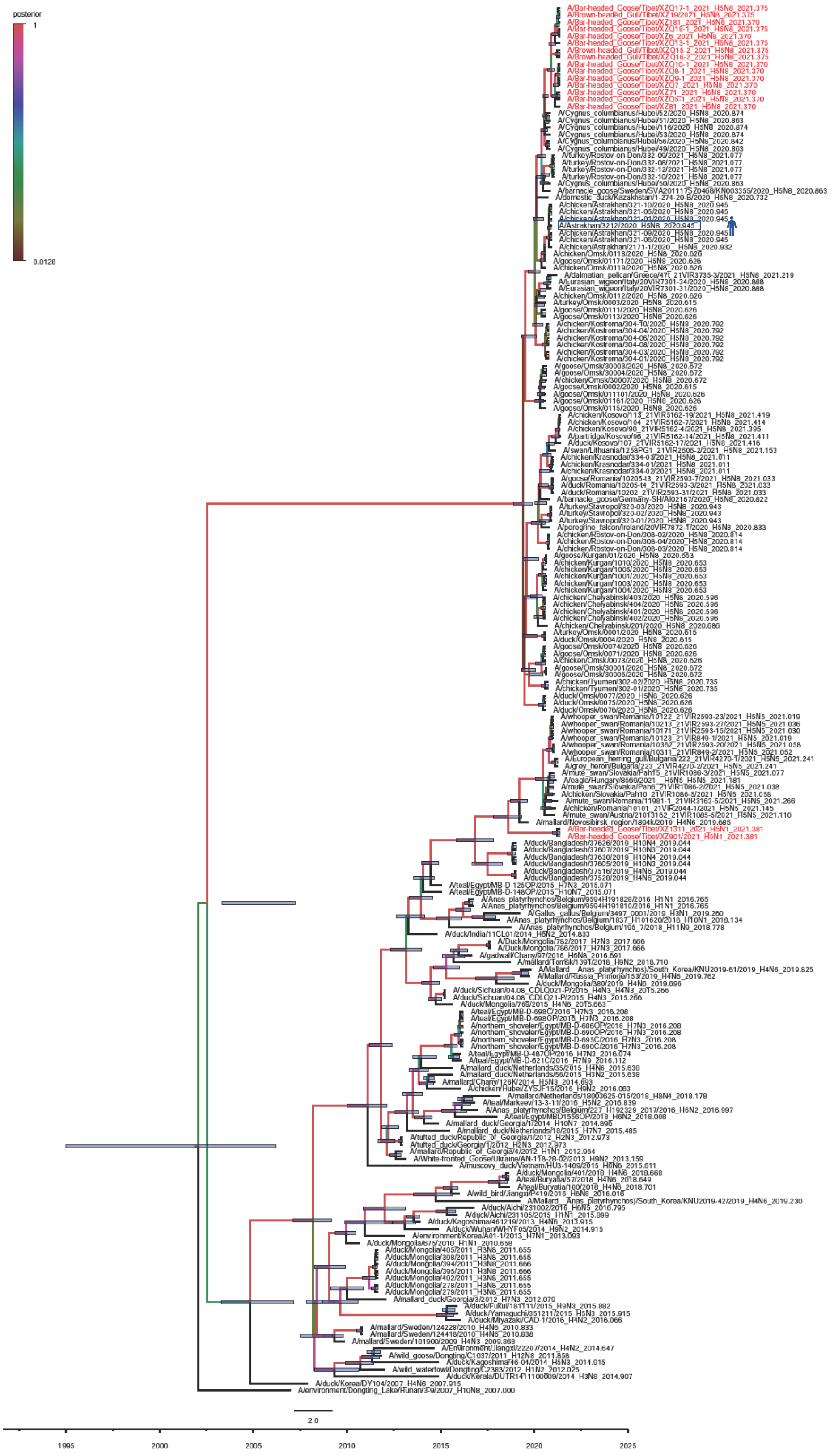

Appendix Figure 2B. PB1

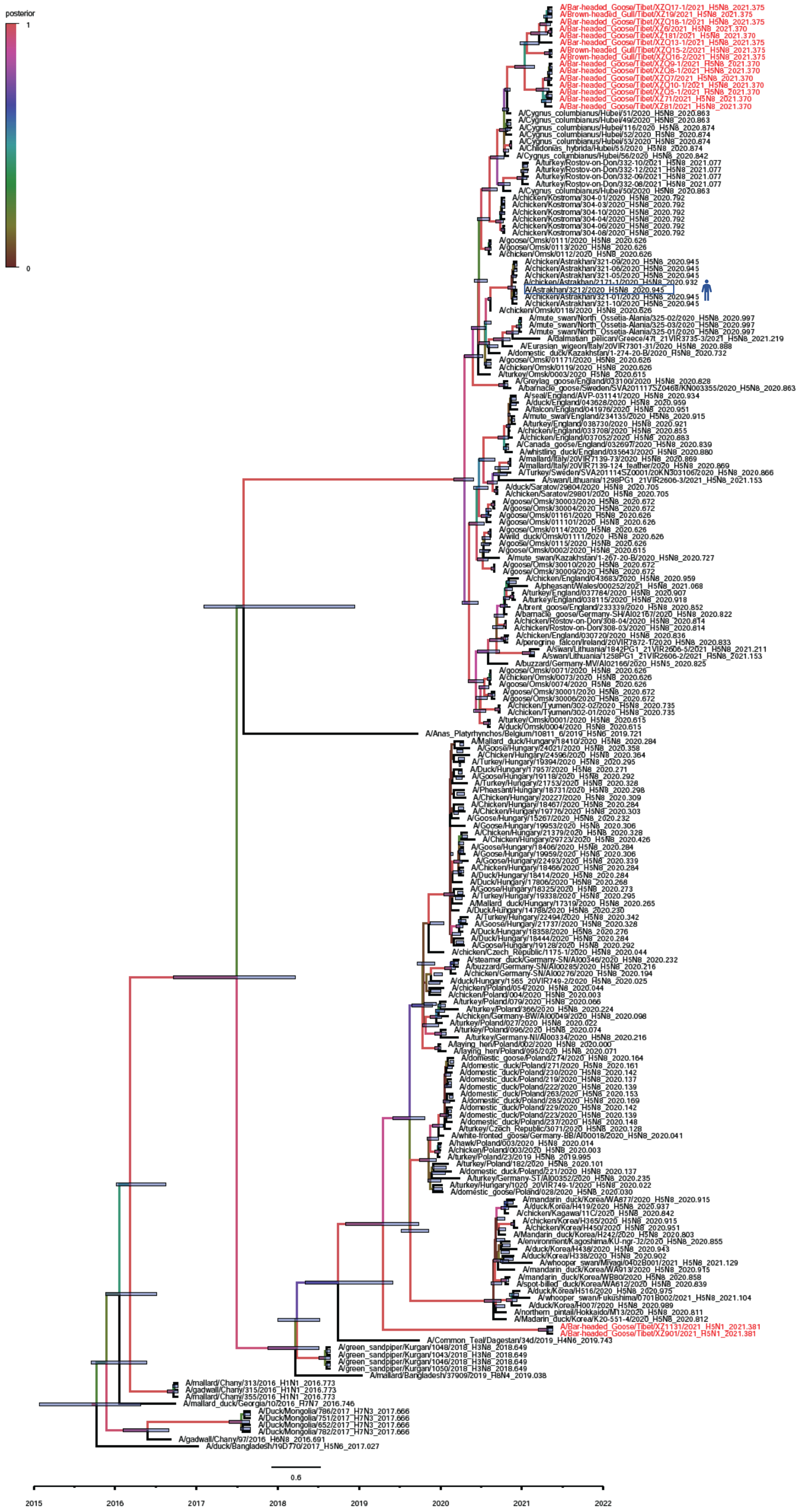

Appendix Figure 2C. PA

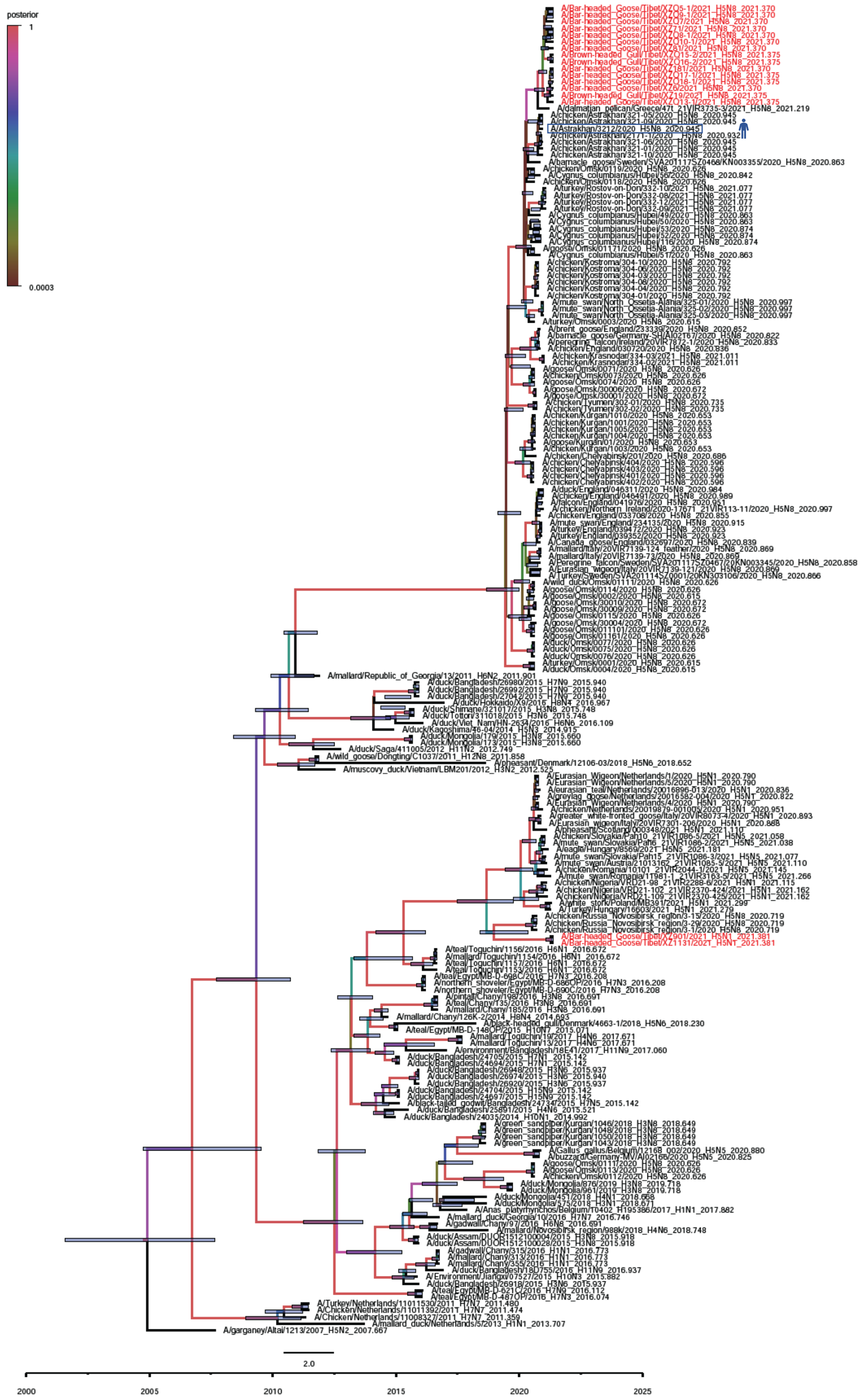

Appendix Figure 2D. NP

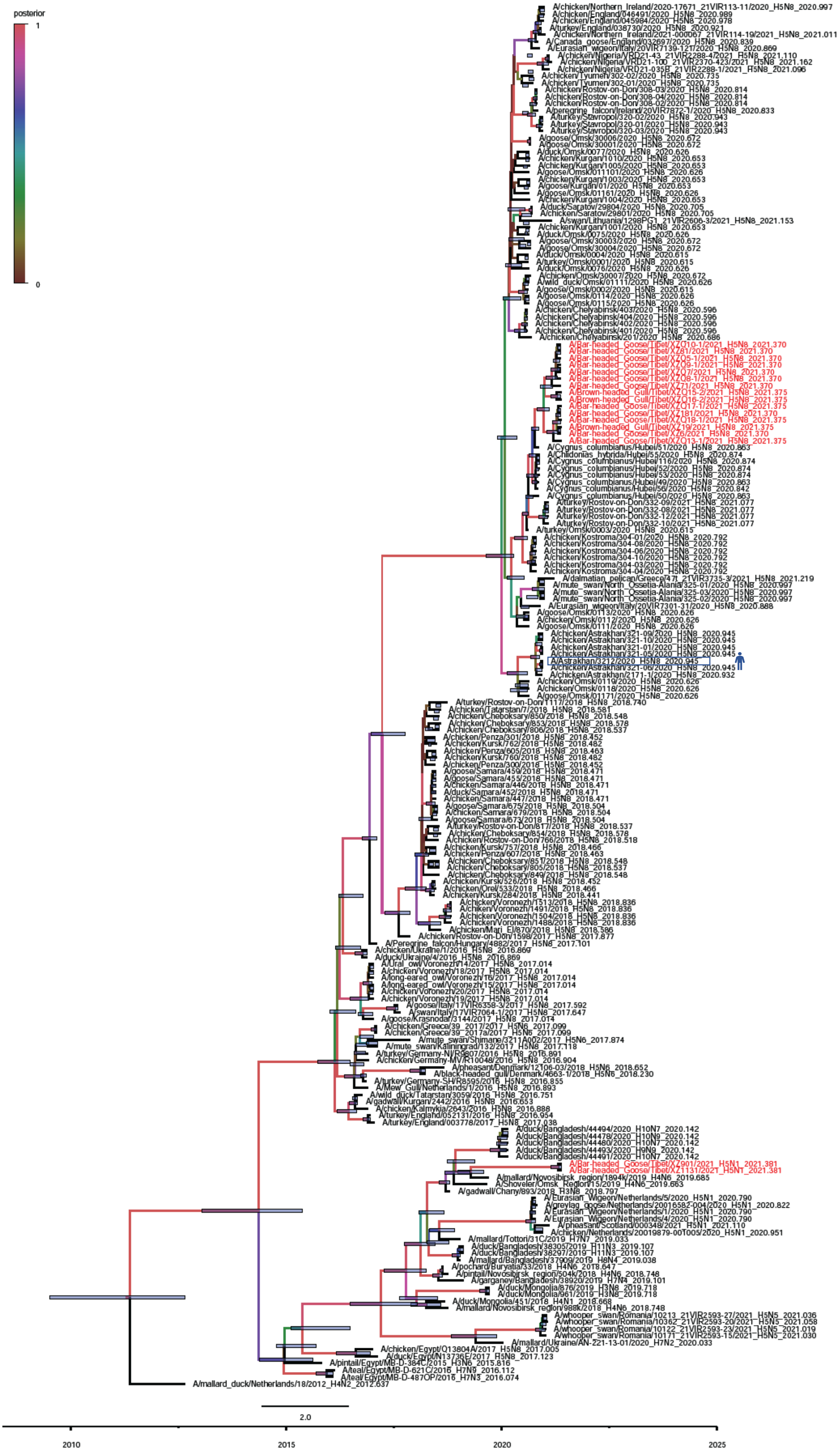

Appendix Figure 2E. NS

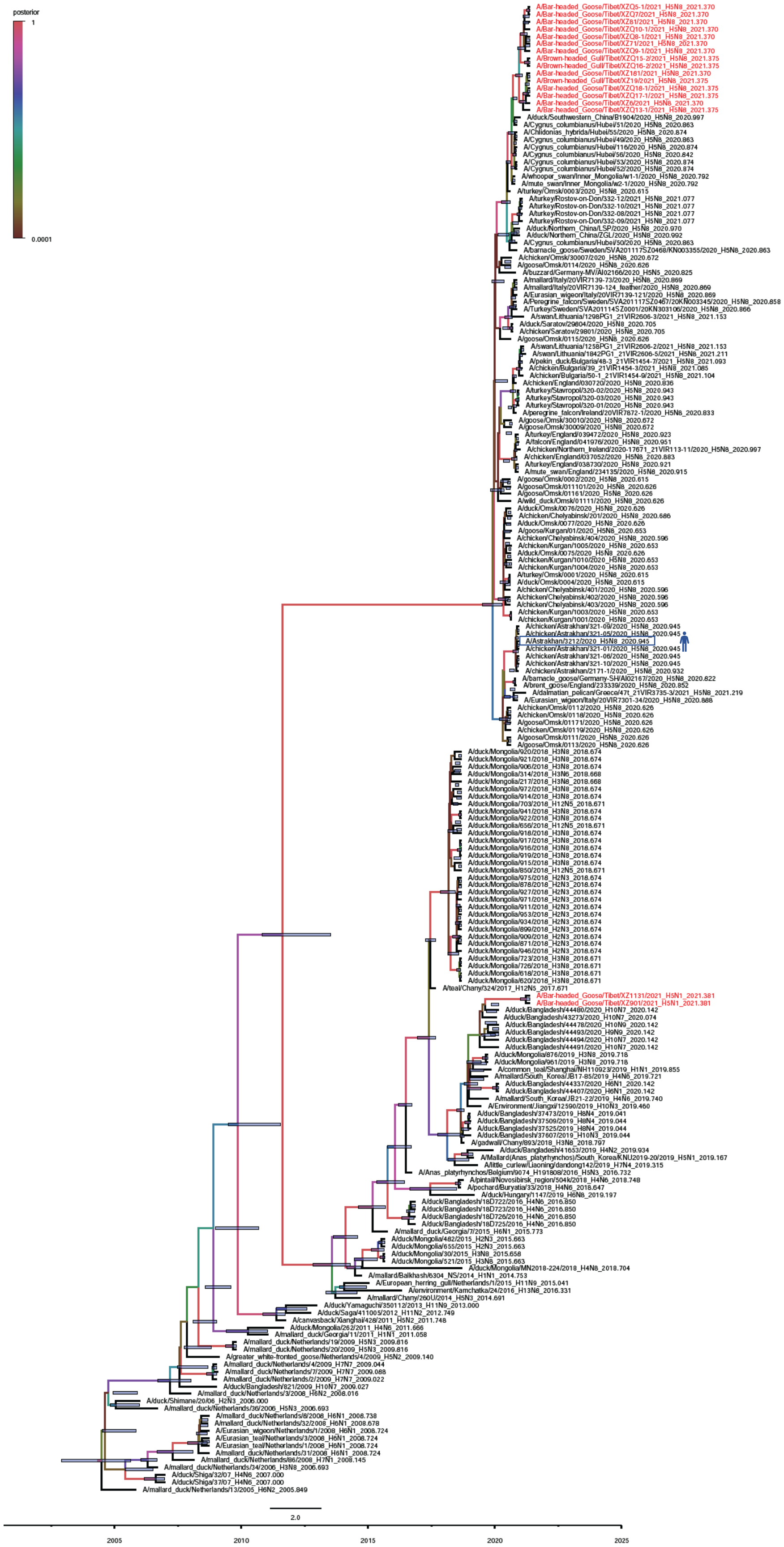

Appendix Figure 2F. M

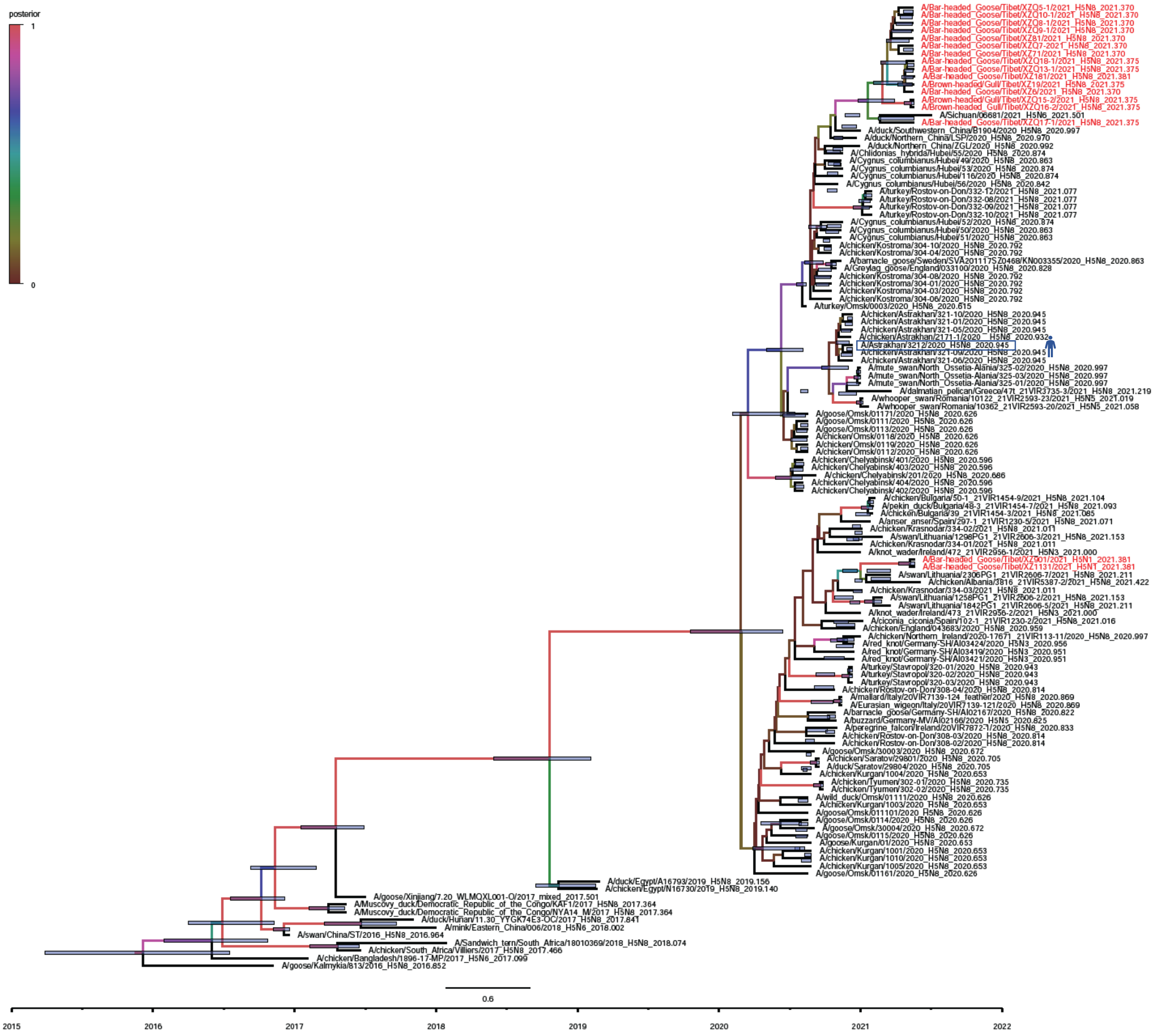

Appendix Figure 2G. N8

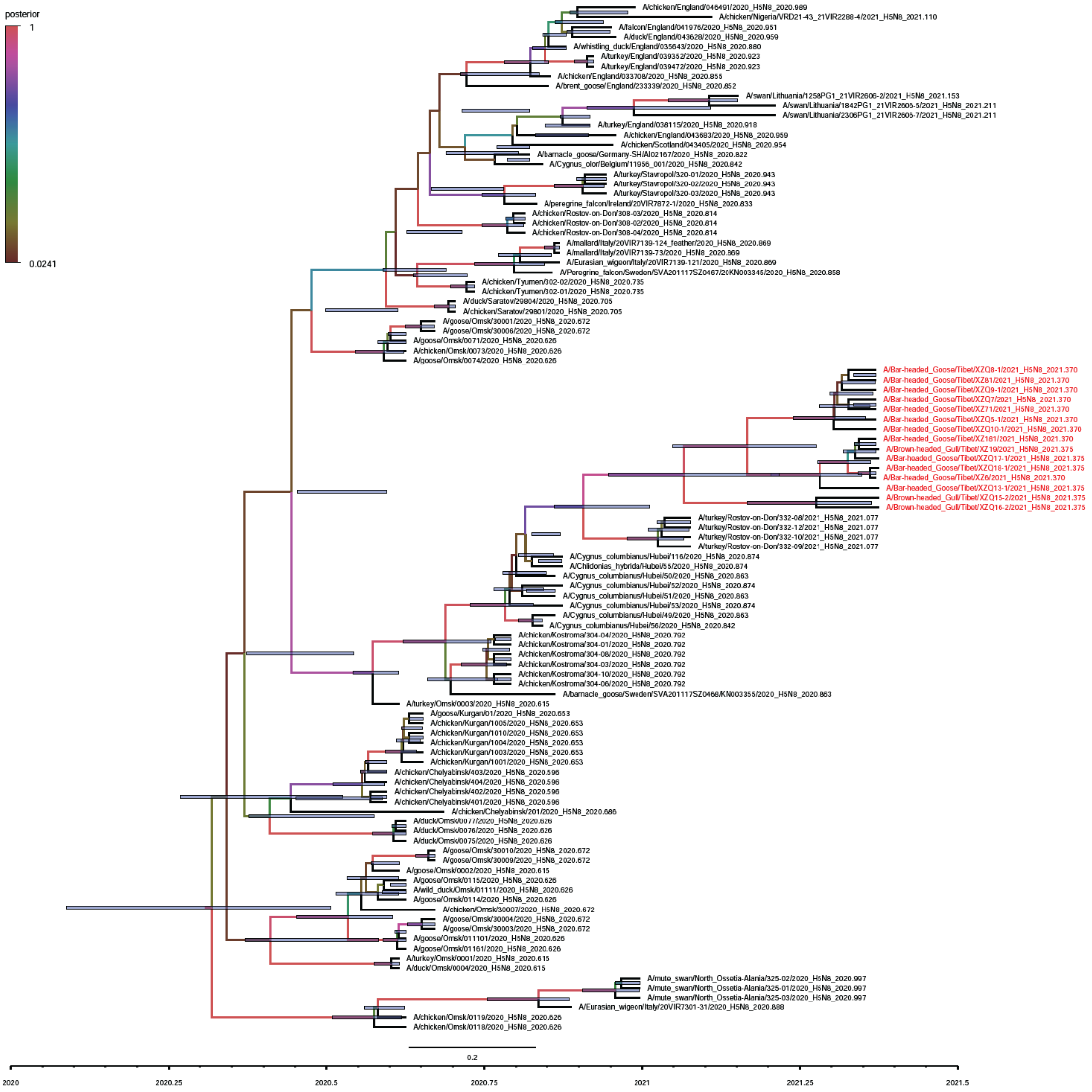

Appendix Figure 2H.N1

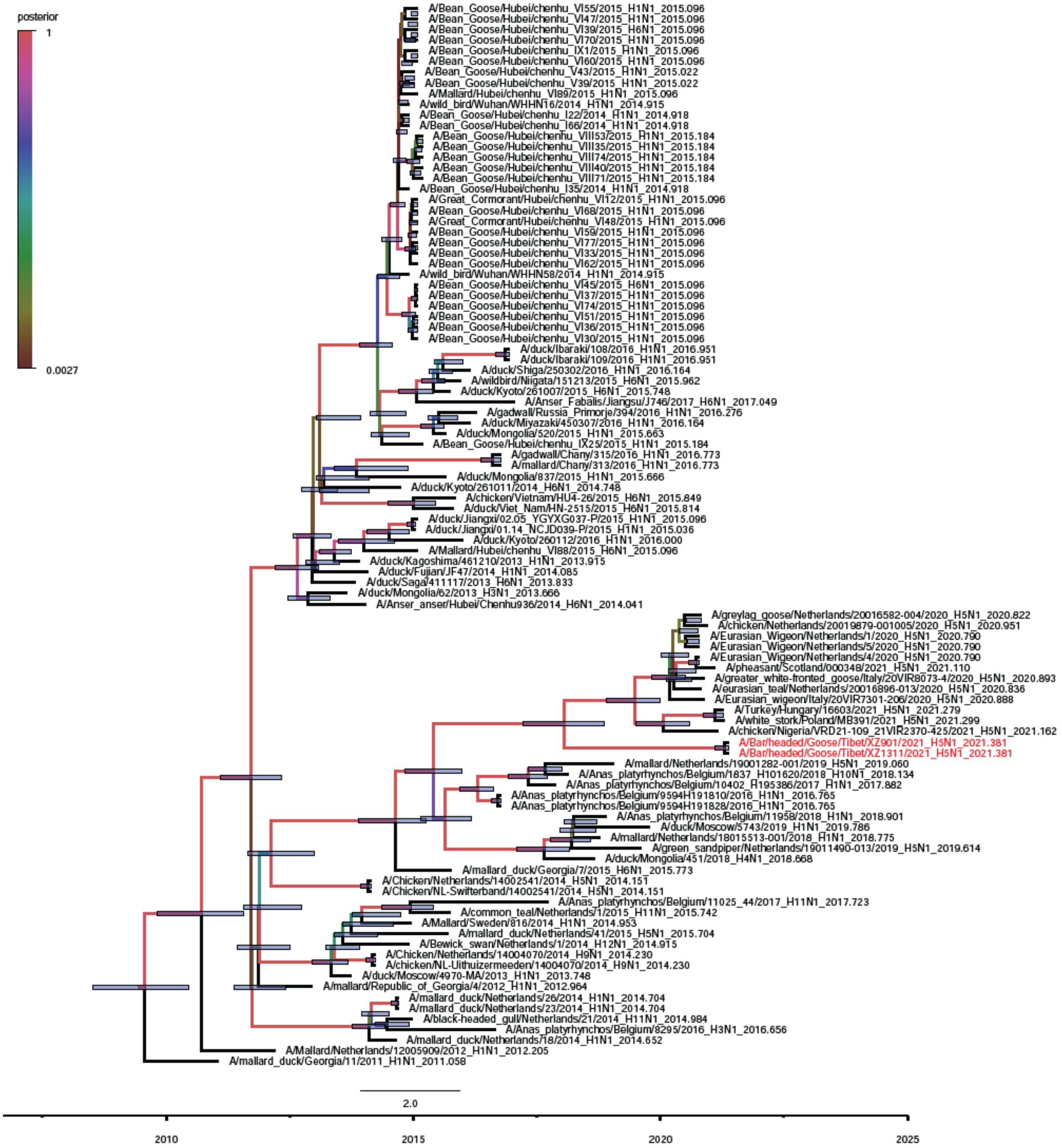

Appendix Figure 2I. H5

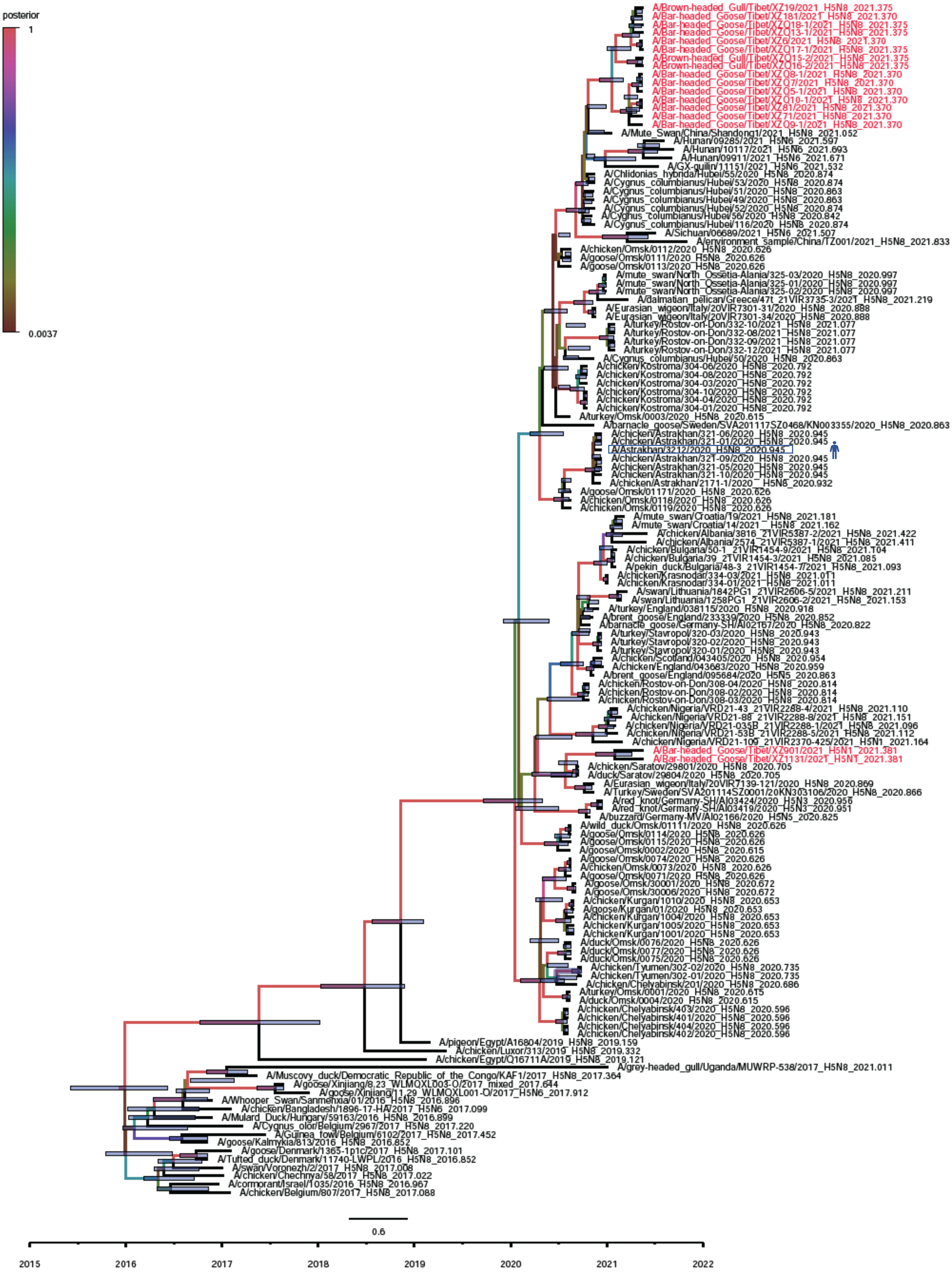

Supplement: SUPPLEMENTAL FILE 1 — Supplemental material. Download spectrum.00643-22-s001.pdf, PDF file, 4.7 MB [file spectrum.00643-22-s001.pdf]
